# Organic anion transporting polypeptides OATP1B1 and OATP1B3 and their genetic variants influence the pharmacokinetics and pharmacodynamics of raloxifene

**DOI:** 10.1186/1479-5876-10-76

**Published:** 2012-04-25

**Authors:** Tina Trdan Lušin, Bruno Stieger, Janja Marc, Aleš Mrhar, Jurij Trontelj, Andrej Zavratnik, Barbara Ostanek

**Affiliations:** 1 Department of Biopharmacy and Pharmacokinetics, Faculty of Pharmacy, University of Ljubljana, Aškerčeva 7, 1000, Ljubljana, Slovenia; 2 Department of Clinical Pharmacology and Toxicology, University Hospital Zürich, Ramistrasse 100, 8091, Zürich, Switzerland; 3 Department of Clinical Biochemistry, Faculty of Pharmacy, University of Ljubljana, Aškerčeva 7, 1000, Ljubljana, Slovenia; 4 Department of Endocrinology and Diabetology, University Medical Centre, Ljubljanska 5, 2000, Maribor, Slovenia

**Keywords:** Raloxifene, Raloxifene diglucuronide, SLCO1B1, SLCO1B3, Osteoporosis, LC/MS/MS

## Abstract

**Background:**

Raloxifene, a selective estrogen receptor modulator, exhibits quite large and unexplained interindividual variability in pharmacokinetics and pharmacodynamics. The aim of this study was to determine the role of organic-anion transporting polypeptides OATP1B1 and OATP1B3 and their genetic variants in the pharmacokinetics and pharmacodynamics of raloxifene.

**Methods:**

To test the role of OATP1B1 and OATP1B3 transporters on hepatic uptake of raloxifene and its metabolites an *in vitro* model of Chinese Hamster Ovary cells expressing OATP1B1 or OATP1B3 was employed. The influence of OATP1B1 and OATP1B3 genetic variants on *in vivo* pharmacokinetics and pharmacodynamics was evaluated in 53 osteoporotic postmenopausal women treated with raloxifene.

**Results:**

Our *in vitro* results showed that raloxifene and two of the three metabolites, raloxifene-4'-β-glucuronide (M2) and raloxifene-6,4'-diglucuronide (M3), interact with OATP1B1 and OATP1B3. Higher M3 and total raloxifene serum concentrations in patients correlated with lower serum levels of bone resorption marker, serum C-terminal telopeptide fragments of type I collagen, indicating a higher antiresorptive effect of raloxifene. Higher concentrations of M2 correlated with higher increase of lumbar spine bone mineral density supporting the raloxifene vertebral fracture specific protection effect. Finally, raloxifene, M3 and total raloxifene serum concentrations were significantly higher in patients with *SLCO1B1 c.*388A > G polymorphism and *1b haplotype implicating a considerable genetic effect on pharmacokinetics and pharmacodynamics of raloxifene.

**Conclusions:**

These findings indicate that *SLCO1B1 c.*388A > G polymorphism could play an important role in pharmacokinetics and pharmacodynamics of raloxifene.

## Background

Raloxifene is a selective estrogen receptor modulator which is approved worldwide for the prevention and treatment of postmenopausal osteoporosis and for the prevention of breast cancer in postmenopausal women. Raloxifene treatment reduces the risk for vertebral fractures [1], increases bone mineral density of lumbar spine (BMD-LS) and of femoral neck (BMD-FN) [2], decreases serum concentrations of bone turnover markers [3,4], stabilizes quantitative ultrasound parameters (QUS) [5,6] and also reduces the risk of invasive breast cancer in postmenopausal women [7]. Beside its beneficial effects on bone and breast tissue, raloxifene decreases total cholesterol, LDL-cholesterol, fibrinogen, lipoprotein(a), high-sensitivity C-reactive protein, homocysteine and cell adhesion molecules levels [8-12]. In postmenopausal women, the favourable effects on cardiovascular risk factors do not seem to translate overall into cardio-protection [13] except in those less than 60 years of age, where a lower incidence of coronary events is observed [14].

Raloxifene is subjected to an extensive first-pass metabolism: 60% of the peroral dose is absorbed but only 2% of unchanged drug reaches the systemic circulation [15]. First-pass metabolism occurs mainly due to conjugation by UDP-glucuronosyltransferases to raloxifene glucuronides, raloxifene-6-β-glucuronide (M1), raloxifene-4'-β-glucuronide (M2) [16] and to raloxifene-6,4'-diglucuronide (M3) [17]. The glucuronides show very little affinity for the estrogen receptors. However, these metabolites can be readily reconverted to active raloxifene in various organs and hence contribute significantly to raloxifene action [18]. Raloxifene, especially in the form of glucuronides, is excreted into bile and completes the enterohepatic cycle and this prolongs its biological half-life to 28 h [15]. Raloxifene is subject to large inter- and intra-individual variability of its clearance and volume of distribution [17] and its pharmacokinetics are still not completely understood, especially the role of transport systems mediating cellular uptake of raloxifene and its metabolites remain to be elucidated. Organic anion-transporting polypeptides (OATP) appear to be particularly important in the disposition of many drugs in clinical use today. Specifically, the liver-enriched OATP1B subfamily members OATP1B1 and OATP1B3 located at the basolateral membrane of hepatocytes exhibit a broad substrate specificity and the ability to transport different drugs [19]. A number of single nucleotide polymorphisms (SNPs) have been found within *SLCO1B1* and/or *SLCO1B3* genes encoding OATP1B1 and OATP1B3 proteins, respectively and were shown to influence the pharmacokinetics and/or pharmacodynamics of many drugs [20-25]. As liver is the main organ governing systemic clearance of raloxifene, the aim of the present study was to identify the role of OATP1B1 and OATP1B3 transporters in hepatic uptake of raloxifene species and to investigate the influence of *SLCO1B1* and *SLCO1B3* genetic polymorphisms on pharmacokinetics and pharmacodynamics of raloxifene in women with postmenopausal osteoporosis.

## Materials and methods

### Chemicals

Radiolabeled ^3^ H] estrone-3-sulfate (E-3-S) was obtained from PerkinElmer Life Sciences (Boston, MA). Cell culture reagents, beta-glucuronidase from *Helix pomatia,* E-3-S, raloxifene hydrochloride and haloperidol were from Sigma Aldrich Chemie (Deisenhofen, Germany). Chinese Hamster Ovary (CHO) cells stably transfected with *SLCO1B1* or *SLCO1B3* are described in Treiber et al. [26] and Gui et al. [27]. Raloxifene metabolites M1, M2 and M3 were synthesised by incubating raloxifene hydrochloride with Streptomyces sp. ATCC 55043 [28] followed by chromatographic purification and lyophilisation. Purity and identification were checked by high performance liquid chromatography (HPLC) and liquid chromatography tandem mass spectrometry (LC-MS/MS). Stock solutions of raloxifene, M1, M2 and M3 were prepared in dimethyl sulfoxide (DMSO).

### Transport experiments in CHO cells

CHO cells were grown at 37°C in a humidified 5% CO_2_ atmosphere in Dulbecco's Modified Eagle Medium (DMEM) supplemented with 10% FBS, 50 μg/mL L-proline, 100 U/mL penicillin and 100 μg/mL streptomycin. For OATP1B1 or OATP1B3 expressing CHO cells the medium was supplemented with geneticin (100 μg/mL). For transport assays, cells were split from a confluent flask at 40,000 cells per dish on 3 cm dishes from Corning (NY, USA) and 48 h later the medium was replaced with a medium containing 5 mM sodium-butyrate to induce nonspecific gene expression [29]. After another 24 h, the cells were 80 to 90% confluent, and transport experiments were preformed as described in Leuthold et al. [30]. Briefly, cells were rinsed three times with pre-warmed (37°C) uptake buffer (116.4 mM NaCl, 5.3 mM KCl, 1 mM NaH_2_PO_4_, 0.8 mM MgSO_4_, 5.5 mM D-glucose and 20 mM Hepes/Tris pH 7.4). The transport experiment was started by adding 1 mL of uptake buffer containing 0.3 μCi/mL of ^3^ H]E-3-S in 0.5 μM E-3-S in the absence or presence of inhibitors: raloxifene (10 μM), M1 (10 μM), M2 (10 μM), M3 (4 μM) and indocyanine green (ICG, 5 μM) as a positive control for inhibition [31]. After 0 and 5 min, the uptake solution was aspirated, the cells rinsed four times with 2 mL of ice-cold uptake buffer and solubilised with 1 mL of 1% Triton X-100. Aliquots were used for liquid scintillation counting and determination of protein concentration. Uptake was calculated by first subtracting the 0 min time point and second correcting the uptake of the CHO-expressing cells by the uptake obtained in CHO wild type cells. Each experiment was performed on four parallels.

### Study participants

A total of 57 Caucasian postmenopausal female patients with osteoporosis were enrolled in the study. The patients were selected according to the following inclusion criteria: >5 years of menopause, aged <70 years, presence of osteoporosis, defined as low BMD (T score < −2.5 SD) or radiographically apparent vertebral, femoral or radius fracture. The exclusion criteria were a history of venous thromboembolic or malignant disease, serious renal impairment, abnormal hepatic function, smoking, osteoporosis therapy, lipid lowering or glucocorticoid treatment and estrogen replacement therapy within previous 6 months.

### Study protocol

Written informed consent was obtained from each individual and the study protocol was approved by the Slovenian National Medical Ethics Committee. The patients were treated for 12 months with 60 mg raloxifene per day and were followed in the University Medical Centre (Maribor, Slovenia). Four patients were removed from the study due to not following the study protocol.

At baseline, blood was drawn for measurements of lipids, bone turnover markers and DNA extraction. All investigations were carried out at 8 a.m. after an overnight fast. After the first visit, the participants started with raloxifene 60 mg, cholecalciferol 400 I.U. and calcium carbonate 1000 mg daily. Patients did not get any dietary advice.

Compliance was checked orally at the regular check-ups by a physician three times during the study; after 3, 6 and 12 months of raloxifene therapy. After 12 months, blood was drawn for measurement of bone turnover markers (serum bone-specific alkaline phosphatase (BALP), serum osteocalcin (OC), serum C-terminal telopeptide fragments of type I collagen (CTX)), lipids and concentrations of raloxifene, M1, M2 and M3. The blood was drawn at 8:00 a.m. visit to the hospital which corresponds to approximately 1–2 hours after the last dose. After centrifugation, the serum samples were stored at −86°C until analysis. The BMD of total hip (BMD-HIP), femoral neck (BMD-FN) and lumbar spine L1-L4 (BMD-LS,) and three quantitative ultrasound parameters, heel speed of sound (SOS), broadband ultrasound attenuation (BUA) and quantitative ultrasound index (QUI) were measured at baseline and after 12 months of raloxifene therapy.

### Determination of raloxifene and its metabolites in serum

The method used for determination of M1, M2 and raloxifene had been developed and validated previously in our laboratory and is described in full detail elsewhere [32]. Additionally, a new method for quantification of metabolite M3 that was found in serum was developed and validated. In the present study, the selected reaction monitoring mode (SRM) set at 826 → 474 (*m/z* transition) was used for the quantification of M3 against the calibration curve for raloxifene using a response factor obtained from incubating the samples in a presence of beta-glucuronidase.

The linear range for M3 was from 0.88 nM to 4800 nM, the limit of quantification was 0.88 nM and the limit of detection was 0.039 nM. The intra- and inter- day coefficients of variation were 2.4 and 1.3%, respectively.

TR (total raloxifene) was calculated as a summation of molar concentrations of raloxifene, M1, M2 and M3.

### Determination of pharmacodynamic parameters

#### Bone mineral density (BMD) measurements

BMD-HIP, BMD-FN and BMD-LS were determined by dual energy X-ray absorptiometry (DXA) with a Hologic QDR-2000+ densitometer (Hologic Inc. Waltham, USA).

#### Quantitative ultrasound (QUS) measurements

QUS measurements of the calcaneus were performed on the left heel of all study participants, with the subject in the sitting position, using a Sahara apparatus (Hologic Inc. Waltham, USA). Three QUS parameters of calcaneus were measured: SOS, BUA and QUI. An average of two measurements was calculated.

#### Bone turnover markers measurements

BALP was assessed by IRMA (Tandem®-R Ostase®; Beckman Coulter). OC was measured using a two-site immunoluminometric Elecsys N-MID Osteocalcin assay (Roche Diagnostics, Mannheim, Germany) [33]. CTX was measured using a two-site immunoluminometric Elecsys Beta CrossLaps assay (Roche Diagnostics, Mannheim Germany) [33].

#### Lipids analysis

Serum total cholesterol (HOL), HDL cholesterol, and triglycerides (TG) were measured with enzymatic methods (Olympus AU640 analyzer). LDL cholesterol was calculated by the Fridewald formula [34].

#### Monitoring of adverse effects

The patients reported the possible onset and the severity of the most frequently described adverse effects associated with raloxifene treatment, such as worsening of hot flushes, leg cramps and swelling. No other adverse effects were noted.

### *SLCO1B1* and *SLCO1B3* genotyping

Genomic DNA was isolated from peripheral blood leukocytes by using a FlexiGene DNA kit (Qiagen, Hilden, Germany). Three SNPs in two different genes c.388A > G (rs2306283) and c.521T > C (rs4149056) in the *SLCO1B1* gene and C > G in intron 7 (rs17680137) of the *SLCO1B3* gene were genotyped using validated TaqMan Assays (C__1901697_20, C__30633906_10, C__25766725_10) from Applied Biosystems in an ABI Prism 7000 sequence detection system, under the conditions recommended by the manufacturer (Applied Biosystems, Foster City, CA, USA). To validate our results, a random selection of 10% of the samples was re-genotyped for each SNP, and the results were found to be reproducible with no discrepancies noted.

### Statistical analysis

The Shapiro-Wilk goodness-of-fit test was used to determine the normality of data distribution and the Levene’s test was used to test the homogeneity of variances prior to the ANOVA and the *t*-test. A square root transformation was applied to the concentrations of raloxifene, M1, M2, M3 and TR in order to obtain a normal data distribution. The Bonferroni post hoc test was used to compare the means from genotype groups for each polymorphism. The calculation of the percentage change of the pharmacodynamic parameters (PD) during treatment was calculated according to Eq.1.

(1) $$\Delta PDparameter=(\frac{PDparameter_{t=0}-PDparameter_{t=12months}}{PDparameter_{0}})\times 100\%$$

A Pearson’s correlation was used for the calculation of associations between the serum concentrations of raloxifene species and the percentage changes of pharmacodynamic parameters. To test the influence of genotype on raloxifene species concentration levels and on percentage change of pharmacodynamic parameters during treatment, the parametric *t*-test or ANOVA were applied. The Hardy-Weinberg equilibrium was tested for each polymorphism using the *χ*^2^ test. The significance criterion (α) was set at p < 0.05. Data analyses were performed by PASW 18 software (IBM company, Illinois, Chicago). Program PHASE version 2 was used for reconstructing haplotypes from population data [35].

## Results

### Interaction of raloxifene and its three metabolites with OATP1B1 and OATP1B3

**Figure 1:**
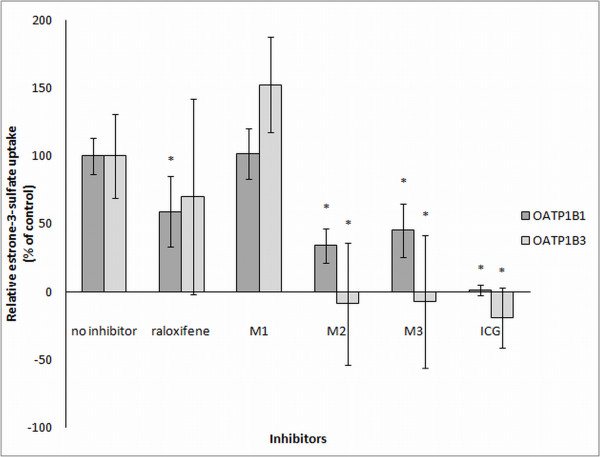
**Inhibitory effects of raloxifene species on active uptake of estrone-3-sulfate into CHO cells expressing OATP1B1 or OATP1B3.** Raloxifene-6-β-glucuronide (M1), raloxifene-4'- β -glucuronide (M2), raloxifene-6,4'-diglucuronide (M3), indocyanine green (ICG), estrone-3-sulfate (E-3-S).E-3-S was used at concentration 0.5 μM, and cells were incubated with substrate and inhibitor for 5 minutes. The results are shown as percentage of control uptake measured in the absence of inhibitor. Each column represents the mean ± SD (n = 4) and (*) indicates a significant difference from the control (p < 0.05).

The interaction potential of raloxifene and its metabolites M1, M2 and M3 with the investigated transporters was tested by observing the inhibition of OATP1B1 and OATP1B3 mediated transport of E-3-S. The concentrations of raloxifene, M1 and M2 used for the experiment were 10 μM, except for M3 that was 4 μM, because the available amount of M3 was very low. Figure 1 shows that raloxifene significantly inhibited OATP1B1 and that M2 and M3 significantly inhibited OATP1B1 and OATP1B3 mediated transport of E-3-S, respectively. Interestingly, M1 did not interact with either OATP, while ICG as a positive control for inhibition as expected completely abolished transport activity of OATP1B1 and OATP1B3. *In vitro* findings strongly indicate that OATP1B1 and OATP1B3 may be involved in raloxifene disposition.

### Patients' baseline characteristics and genotype frequencies

The baseline characteristics of 53 postmenopausal women with osteoporosis enrolled in the study are presented in Table 1.

**Table 1:** Summary of baseline characteristics of 53 Caucasian postmenopausal women

	**mean (SD)**	**Range**
**Age (year)**	59.7 (6.2)	39.0 – 69.0
**Years postmenopausal (year)**	13.1 (6.9)	5.0 – 38.0
**BMI (kg/m**^**2**^**)**	25.6 (3.4)	18.9 – 34.9
**SBP (mmHg)**	143.4 (17.8)	105.0 – 190.0
**DBP (mmHg)**	85.5 (10.0)	60.0 – 105.0
**BALP (μg/L)**	12.2 (5.3)	4.9 – 29.1
**OC (ng/mL)**	33.5 (11.3)	10.0 – 60.3
**CTX (ng/mL)**	0.52 (0.19)	0.12 – 0.89
**BUA (dB/MHz)**	54.8 (11.5)	33.8 – 85.9
**SOS (m/s)**	1,513.5 (21.1)	1,464.4 – 1,562.6
**QUI**	72.0 (12.7)	43.9 – 104.9
**BMD-HIP(g/cm**^**2**^**)**	0.727 (0.096)	0.472 – 0.910
**BMD-FN(g/cm**^**2**^**)**	0.627 (0.076)	0.420 – 0.840
**BMD-LS (g/cm**^**2**^**)**	0.759 (0.071)	0.586 – 0.950
**HOL (mmol/L)**	5.85 (0.78)	4.31 – 7.37
**HDL (mmol/L)**	1.65 (0.31)	1.19 – 2.36
**LDL (mmol/L)**	3.53 (0.67)	2.17 – 4.81
**TG (mmol/L)**	1.50 (0.72)	0.67 – 4.03

BMI, body mass index; SBP, systolic blood pressure; DBP, diastolic blood pressure; BALP, serum bone-specific alkaline phosphatase; OC, serum osteocalcin; CTX, serum C-terminal telopeptide fragments of type I collagen; BUA, broadband ultrasound attenuation; SOS, heel speed of sound; QUI, quantitative ultrasound index; BMD-HIP, bone mineral density of total hip; BMD-FN, bone mineral density of femoral neck; BMD-LS, bone mineral density of lumbar spine.

Based on the *in vitro* results, the SNPs of *SLCO1B1* c.388A > G, *SLCO1B1* c.521T > C and *SLCO1B3* int7C > G [36] were analyzed. Genotype and haplotype frequencies are presented in Table 2. In case of the *SLCO1B1* c.521T > C polymorphism there were no participants with the CC genotype, so we compared only the TT and TC genotypes. As there was only one participant with the GG genotype for *SLCO1B3* int7C > G polymorphism, we combined the patients with GG and CG genotypes into the non-CC group. The distribution of genotypes did not deviate significantly from the Hardy-Weinberg equilibrium (p > 0.05).

**Table 2:** **Genotype and haplotype frequencies of*SLCO*polymorphisms in 53 postmenopausal patients with osteoporosis**

***SLCO *variant**	**genotype**	**number of patients**	**frequency (%)**
***SLCO1B1*c.388A > G**	AA	14	26.4
	AG	28	52.8
	GG	11	20.8
***SLCO1B1*c.521T > C**	TT	35	66.0
	TC	18	34.0
	CC	0	0
***SLCO1B3*int7C > G**	CC	36	67.9
	CG	16	30.2
	GG	1	1.9
***SLCO1B1*haplotype**	**number haplotype copies**		
*1a (reference haplotype)	0	11	20.8
	1	30	56.6
	2	12	22.6
*1b (c.388A > G)	0	24	45.3
	1	24	45.3
	2	5	9.4
*5 (c.521T > C)	0	51	96.2
	1	2	3.8
	2	0	0
*15 (c.388A > G and c.521T > C)	0	37	69.8
	1	16	30.2
	2	0	0

In case of haplotypes, only the influence of haplotypes *1a, *1b and *15 was tested, but not *5, because there were not enough patients with this haplotype.

### Serum concentrations of raloxifene species in relation to genotypes and haplotypes after 12 months of raloxifene treatment

**Figure 2:**
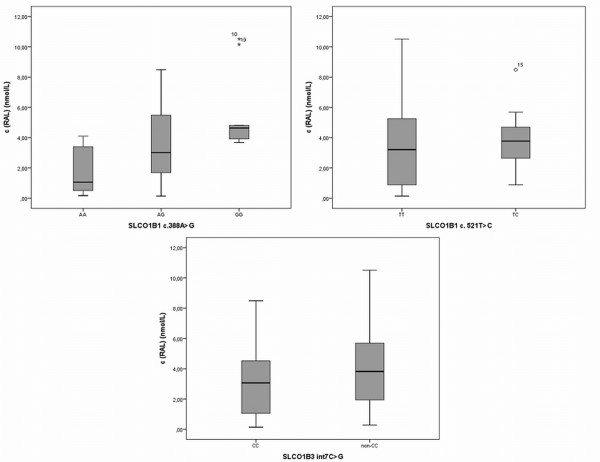
**Raloxifene concentrations according to the *SLCO1B1 *c.388A > G (rs2306283), *SLCO1B1 *c.521T > C, and *SLCO1B3 *int7C > G polymorphisms.**

A high variability in serum concentrations of all measured raloxifene species (M1, M2, M3, raloxifene, TR) was observed between patients after 12 months of raloxifene treatment. Mean serum concentrations (SD) were 70.1 (59.0), 315.6 (276.3), 510.0 (580.3), 3.6 (2.6) and 899.3 (829.7) for M1, M2, M3, raloxifene and TR, respectively (Figure 2).

Statistical analyses showed a strong influence of *SLCO1B1* c.388A > G polymorphism on the concentrations of raloxifene, M3 and TR. The levels of raloxifene, M3 and TR were increasing from AA to GG individuals. The same trend was observed for M1 and M2 concentrations, but the differences were not statistically significant (Table 3). *SLCO1B1* c.388A > G polymorphism explains 26.3%, 22.8% and 20.8% of variability of M3, RAL, and TR concentrations, respectively.

**Table 3:** **Summary of concentrations of raloxifene species by*SLCO1B1*and*SLCO1B3*polymorphisms**

	**Genotype**									
	***SLCO1B1*c.388A > G**				***SLCO1B1*c.521T > C**			***SLCO1B3*int7C > G**		
	**AA**	**AG**	**GG**	**p value (ANOVA)**	**TT**	**TC**	**p value (*t*-test)**	**CC**	**non-CC**	**p value (*t*-test)**
**c (M1) (nmol/l)**	60 (19)	63 (12)	99 (19)	0.143	65 (11)	79 (15)	0.400	68 (10)	75 (19)	0.830
**c (M2) (nmol/l)**	268 (90)	288 (53)	439 (101)	0.225	254 (43)	431 (85)	0.076	313 (51)	321 (77)	0.959
**c (M3) (nmol/l)**	266 (88)	395 (73)	1073 (305)	**0.001**^**a**^	545 (130)	446 (98)	0.917	411 (53)	715 (246)	0.339
**c (RAL) (nmol/l)**	1.9 (0.5)	3.5 (0.5)	5.6 (0.9)	**0.004**^**b**^	3.5 (0.6)	3.8 (0.5)	0.308	3.2 (0.4)	4.5 (0.8)	0.166
**c (TR) (nmol/l)**	596 (182)	750 (130)	1617 (381)	**0.009**^**c**^	867 (174)	960 (167)	0.519	795 (107)	1115 (321)	0.428

The values of concentrations are presented as means (standard errors).

^a^Bonferoni post hoc test (ANOVA), p = 0.004 for AG vs. GG,p =0.001 for AA vs. GG.

^b^Bonferoni post hoc test (ANOVA), p = 0.003 for AA vs. GG.

^c^Bonferoni post hoc test (ANOVA), p = 0.014 for AA vs. GG,p =0.020 for AG vs. GG.

M1, raloxifene-6-β-glucuronide; M2, raloxifene-4'- β –glucuronide; M3, raloxifene-6,4'- di –glucuronide; RAL, raloxifene; TR, total raloxifene.

On the other hand, no statistically significant association of serum raloxifene, M1, M2, M3 and TR concentrations with *SLCO1B1* c.521T > C and *SLCO1B3* int7C > G polymorphisms was observed. However, a trend towards lower concentrations of raloxifene and its metabolites in wild type individuals compared to TC group (*SLCO1B1* c.521T > C) and to non-CC group (*SLCO1B3* int7C > G) was observed, but did not reach statistical significance (Table 3).

Additionally, the statistical analysis of raloxifene species levels according to the number of copies of haplotypes for *SLCO1B1* showed that patients with more copies of *1b haplotype have a higher concentration of M3, raloxifene and TR. It was found out that *1b haplotype explains 32.3%, 18.6% and 17.7% of variability of M3, RAL, and TR concentrations, respectively.

On the contrary, a contribution of *15 haplotype copies to the concentrations of raloxifene species was not observed. When testing the influence of the wild type haplotype *1a on raloxifene species levels, significance was observed for M3, raloxifene and TR. More copies of *1a haplotype correspond to lower raloxifene species levels, which is consistent with the results for *1b haplotype (Table 4).

**Table 4:** Summary of concentrations of raloxifene species by haplotype

	***SLCO1B1*haplotype**										
	*1b				*15			*1a			
**No. of haplotype copies**	**0**	**1**	**2**	**p value (ANOVA)**	**0**	**1**	**p value (*t*-test)**	**0**	**1**	**2**	**p value (ANOVA)**
**M1 (nmol/l)**	61.8 (12.0)	72.9 (15.0)	92.6 (17.3)	0.437	66.0 (11.0)	78.4 (16.0)	0.497	99.4 (19.0)	64.3 (11.5)	55.9 (20.5)	0.125
**M2 (nmol/l)**	334.1 (72.0)	281.3 (57.1)	412.3 (122.9)	0.625	264.2 (42.5)	422.1 (90.8)	0.138	439.2 (101.7)	298.7 (52.3)	231.7 (93.0)	0.18
**M3 (nmol/l)**	305.8 (60.2)	482.7 (87.1)	1,572.3 (616.9)	**0.0004**^**a**^	533.1 (125.9)	462.3 (81.8)	0.954	1,072.7 (305.2)	388.1 (70.2)	273.3 (99.0)	**0.001**^**d**^
**RAL** **(nmol/l)**	2.7 (0.5)	3.7 (0.5)	7.2 (1.8)	0.016^b^	3.5 (0.5)	3.8 (0.5)	0.343	5.6 (0.9)	3.5 (0.5)	1.7 (0.6)	0.002^e^
**TR (nmol/I)**	704.4 (137.6)	840.6 (154.0)	2,084.4 (751.6)	0.02^c^	866.8 (168.1)	966.9 (380.7)	0.549	1,616.9 (380.7)	754.6 (124.6)	592.6 (202.9)	0.007^f^

The values of concentrations are presented as means (standard errors).

^a^Bonferoni test (ANOVA), p = 0.004 for 1 vs. 2 copies, p =0.0003 for 0 vs. 2 copies.

^b^ Bonferoni test (ANOVA), p = 0.014 for 0 vs. 2 copies.

^c^Bonferoni test (ANOVA), p = 0.017 for 0 vs 2 copies.

^d^Bonferoni test (ANOVA), p = 0.004 for 0 vs. 1 copies, p =0.008 for 0 vs. 2 copies.

^e^Bonferoni test (ANOVA), p = 0.003 for 0 vs. 2 copies.

^f^Bonferoni test (ANOVA), p = 0.016 for 0 vs. 1 copies, p =0.019 for 0 vs. 2 copies.

M1, raloxifene-6-β-glucuronide; M2, raloxifene-4'- β –glucuronide; M3, raloxifene-6,4'- di –glucuronide; RAL, raloxifene; TR, total raloxifene.

### Drug-drug pharmacokinetic interactions via OATPs

Drug-drug pharmacokinetic interactions via OATPs have been evaluated on the basis of concomitant medication data of the patients. There were only few patients receiving known substrates or inhibitors of OATP1B1 and/or OATP1B3 such as diclofenac [37], atorvastatin [22] and enalapril [38]. Regarding the possibility of drug-drug pharmacokinetic interactions via OATPs, the results from the ANOVA test show that there were no statistically significant influences of concomitantly administered OATP substrates on raloxifene species concentration levels. Vice-versa interactions cannot be ruled out, however the concomitant medications concentration levels were not monitored, therefore no conclusions can be made.

### Correlation of raloxifene species serum concentrations with the percentage change of pharmacodynamic parameters

Pearson’s correlation analysis gave significant results for correlation of M3 with Δ CTX (r = − 0.324, N = 40, p = 0.044, two-tailed), TR with Δ CTX (r = − 0.376, N = 40, p = 0.017, two-tailed) and M2 with the percentage change of bone mineral density of lumbar spine (Δ BMD-LS) (r = 0.320, N = 40, p = 0.044, two-tailed).

### Impact of OATP1B1 and OATP1B3 genotypes and haplotypes on the pharmacodynamic parameters

In all patients after 12 months of raloxifene therapy, all the tested parameters (BALP, OC, CTX, BUA, SOS, QUI, BMD-HIP, BMD-FN, BMD-LS, HOL, HDL, LDL, TG) changed in the same way as previously observed [2-4,6,8,9,11,39-42]. None of the three tested QUS parameters QUI, BUA and SOS showed any significant changes after 12 months of therapy (data not shown).

The relative changes of the selected pharmacodynamic parameters after 12 months of raloxifene treatment were evaluated according to *SLCO1B1* c.388A > G*, SLCO1B1* c.521T > C and *SLCO1B3* int7C > G polymorphisms. Table 5 shows a significant association of genotypes with bone turnover markers BMD-FN, QUI and OC. It was found out that tested polymorphisms explain up to 23% of variability of the significantly changed Δ FN BMD, Δ QUI and Δ OC.

**Table 5:** **Percentage change of pharmacodynamic parameters according to*SLCO1B1*c.388A > G,*SLCO1B1*c.521T > C and*SLCO1B3*int7A > G genotypes and*SLCO1B1*haplotypes after 12 months of raloxifene treatment in 53 postmenopausal women**

**Pharmacodynamic parameter**	**Δ FN BMD (%)**		**Δ QUI (%)**		**Δ OC (%)**			
**genotype**	SLCO1B1 c.388A > G		**SLCO1B1 c.521T > C**		**SLCO1B3 int7C > G**			
	AA	−1.1 (1.5)	TT	2.5 (1.1)	CC	−21.0 (3.5)		
	AG	3.4 (0.6)	TC	−1.6 (1.4)	non CC	−33.5 (3.5)		
	GG	−0.6 (1.1)						
**test/p-value**	ANOVA	**0.001**^**a**^	*t*-test	**0.033**	*t*-test	**0.015**		
**Pharmacodynamic parameter**	**Δ FN BMD (%)**		**Δ CTX (%)**		**Δ SOS (%)**		**Δ QUI(%)**	
**haplotype**	*1a		*1b		*15		*15			
	0 copies	−0.65 (1.1)	0 copies	−20.7 (6.7)	0 copies	0.2 (0.1)	0 copies	2.4 (1.1)		
	1 copy	3.5 (0.6)	1 copy	−24.7 (5.1)	1 copy	−0.3 (0.2)	1 copy	−1.9 (1.6)		
	2 copies	−2.0 (1.6)	2 copies	−56.9 (10.4)						
**test/p-valuetest/p-value**	ANOVA	**0.0002**^**b**^	ANOVA	**0.039**^**c**^	*t*-test	**0.031**	*t*-test	**0.03**		

The values are presented as means (standard errors).

^a^Bonferoni test (ANOVA), p = 0.004 for AA vs. AG, =0.021 for AG vs. GG.

^b^Bonferoni test (ANOVA), p = 0.013 for 0 vs. 1, p = 0.0004 for 1 vs. 2 copies of haplotype*1a.

^c^Bonferoni test (ANOVA), p = 0.035 for 0 vs. 2 copies of haplotype*1b.

Δ FN BMD (%), percentage change of femoral neck bone mineral density; Δ QUI (%), percentage change of quantitative ultrasound index; Δ OC (%), percentage change of osteocalcin; Δ CTX (%), percentage change of serum C-terminal telopeptide fragments of type I collagen; Δ SOS (%), percentage change of heel speed of sound.

Additionally, a statistical analysis of pharmacodynamic parameters according to the *SLCO1B1* haplotype was performed and the significant results are presented in Table 5. *SLCO1B1* haplotypes explained up to 29% of the observed variability of significant pharmacodynamic parameters.

Further, the influence of *SLCO1B1* c.388A > G*, SLCO1B1* c.521T > C and *SLCO1B3* int7C > G polymorphisms on HOL, HDL, LDL and TG was tested but no statistical significance was observed (Table 6 and Table 7).

**Table 6:** **Percentage change of lipids according to*SLCO1B1*c.388A > G,*SLCO1B1*c.521T > C and*SLCO1B3*int7A > G genotypes after 12 months of raloxifene treatment in 53 postmenopausal women**

	**Genotype**									
	**SLCO1B1 c.388A > G**				**SLCO1B1 c.521T > C**			**SLCO1B3 int7 T → G**		
	**AA**	**AG**	**GG**	**p value (ANOVA)**	**TT**	**TC**	**p value (*t*-test)**	**CC**	**non CC**	**p value (*t*-test)**
**Δ HOL (%)**	0.08 (±2.41)	−3.8 (±2.8)	−6.9 (±2.0)	0.368	−4.0 (±1.9)	−2.7 (±3.5)	0.725	−2.3 (±2.1)	−6.1 (±2.7)	0.291
**Δ HDL (%)**	−1.3 (±2.7)	3.0 (±3.2)	−3.9 (±4.0)	0.379	0.7 (±2.5)	0.06 (±3.7)	0.890	1.7 (±2.4)	−2.0 (±3.8)	0.393
**Δ LDL (%)**	2.7 (±3.8)	−6.1 (±3.5)	−10.3 (±3.4)	0.132	−4.9 (±2.5)	−4.9 (±4.7)	0.994	−4.4 (±3.1)	−5.8 (±3.2)	0.781
**Δ TG (%)**	12.9 (±12.8)	−6.7 (±4.2)	21.3 (±12.1)	**0.041**^a^	−1.2 (±6.8)	14.6 (±7.2)	0.124	6.8 (±6.4)	−0.9 (±7.1)	0.455

The values are presented as means (standard errors).

^a^Bonferoni test (ANOVA), no differences.

**Table 7:** **Percentage change of lipids according to*SLCO1B1*haplotypes after 12 months of raloxifene treatment in 53 postmenopausal women**

	**Haplotype**										
	*1b				*15			*1a			
**Number of copies of haplotype**	**0**	**1**	**2**	**p value (ANOVA)**	**0**	**1**	**p value (*t*-test)**	**0**	**1**	**2**	**p value (ANOVA)**
**Δ HOL (%)**	−0.8 (2.9)	−4.8 (2.2)	−10.2 (3.0)	0.228	−3.9 (1.8)	−2.8 (3.7)	0.747	−6.9 (2.0)	−3.8 (2.7)	0.3 (2.6)	0.363
**Δ HDL (%)**	−0.7 (2.9)	3.8 (3.2)	−9.7 (5.3)	0.149	0.5 (2.4)	0.3 (4.0)	0.963	−3.9 (4.0)	2.7 (3.1)	−1.0 (3.0)	0.416
**Δ LDL (%)**	−0.05 (4.01)	−7.8 (2.7)	−13.0 (5.8)	0.140	−4.8 (2.5)	−5.2 (5.0)	0.935	−10.3 (3.4)	−5.9 (3.4)	3.0 (4.1)	0.140
**Δ TG (%)**	8.7 (7.7)	−1.0 (6.4)	7.8 (20.7)	0.626	−0.7 (6.0)	14.5 (7.7)	0.145	21.3 (12.1)	−5.9 (4.2)	12.7 (14.0)	0.051

## Discussion

The aim of our study was to investigate the role of uptake transporters and their genetic variations in pharmacokinetics and pharmacodynamics of raloxifene in postmenopausal osteoporosis.

Our *in vitro* experiments with OATP1B1 and OATP1B3 transfected CHO cell lines showed that M2 and M3 interact with both, OATP1B1 and OATP1B3 transporters, raloxifene interacts only with OATP1B1 and M1 does not interact with any of the studied transporters. This does not diminish the physiological importance of the tested transporters, because as we have demonstrated, M1 *in vivo* concentrations are much lower compared to the concentrations of M2 and M3. A direct transport of raloxifene species into the transfected cell lines could not be measured, because the radiolabelled raloxifene species were not available. In addition, affinities or IC 50s could not be determined due to the very small amount of available raloxifene metabolites.

On the basis of this *in vitro* data we decided to study the effect of *SLCO1B1* and *SLCO1B3* genetic variants on the pharmacokinetics and pharmacodynamics of raloxifene in a group of Caucasian postmenopausal osteoporotic women. Therefore, genotyping of three polymorphisms, *SLCO1B1* c.388A > G, *SLCO1B1* c.521T > C and *SLCO1B3* int7C > G which were previously shown to alter transporter activity of some drugs [20-25] was performed. The *SLCO1B1* c.388A > G polymorphism is responsible for the amino acid substitution (p.N130D) in extracellular loop 2 of the OATP1B1 protein and was shown to affect substrate specificity suggesting that this loop is involved in substrate recognition [43,44]. This polymorphism could be responsible for unchanged, increased or reduced transport activities of OATP1B1 [23,45-47]. On the other hand, it was found that *SLCO1B1* c.521T > C (p.V174A) polymorphism decreases the membrane expression of OATP1B1 protein and consequently lowers its transport activity [48]. Because of the high allelic frequencies in Caucasian population, 41% for c.388A > G and 18% for c.521T > C [49], and influence on transport activities, the c.388A > G and c.521T > C polymorphisms could contribute to the high inter-individual variability in pharmacokinetics and pharmacodynamics of raloxifene. Additionally, a third polymorphism located in intron 7 of *SLCO1B3* gene was studied because of its strong association with unconjugated bilirubin [36], whose levels were found to correlate with those of raloxifene.

Serum concentrations of raloxifene, M1, M2 and M3 were determined using a validated method. To our knowledge, the developed LC/MS/MS method is the first method for detection of M1, M2, M3 and raloxifene. The novelty of this contribution is the observation that the main *in vivo* metabolite of raloxifene is M3. Our *in vivo* study showed that the average raloxifene species serum levels are in ratio of 1 : 19.5 : 87.7 : 141.7, for RAL, M1, M2 and M3, respectively.

Serum levels of raloxifene, M3 and TR increase with the number of *SLCO1B1* c.388A > G mutated alleles. Levels of M1 and M2 were visibly elevated as well, but due to the small sample size and the pronounced data variability, a statistical confirmation of this observation could not be made. Analyses of possible combinations of both *SLCO1B1* gene variants (haplotype) were consistent with the results of individual testing of *SLCO1B1* polymorphisms and showed that haplotype *1b caused higher concentrations of raloxifene, M3 and TR. These results could indicate that the uptake of raloxifene in hepatocytes is lower and consequently, the serum concentrations are higher. Moreover, concentrations of metabolites also increase with the number of mutated alleles.

It is well known that only unconjugated raloxifene can bind with high affinity to estrogen receptors to evoke a pharmacodynamic response [18], although it represents <1% of total serum raloxifene [15]. This small fraction of unconjugated raloxifene found in systemic circulation originates partly directly from the parent compound absorbed from the gut and partly from the systemic cleavage of the circulating glucuronides into raloxifene [17]. Hence, the raloxifene metabolites actually represent transport forms and a depot of active raloxifene. Given the extremely low and highly variable raloxifene concentrations, the serum levels of raloxifene metabolites can be used as a measure of raloxifene exposure.

Our correlation analysis between serum levels of raloxifene species with clinical parameters showed higher levels of M3 and TR in patients with a higher decrease in serum CTX, one of the most reliable bone resorption markers [50], thus implicating a better antiresorptive effect of raloxifene in those patients. In addition, higher M2 concentrations were found to correlate with a greater increase in BMD at lumbar spine (BMD-LS) but not with BMD values at other sites of skeleton. This is in concordance with the already known “site specific” bone effects during raloxifene treatment, which is therefore predominantly used for the prevention of vertebral osteoporotic fractures and not for hip or wrist or any other common osteoporotic fractures. Based on this correlation, the influence of *SLCO1B1* and *SLCO1B3* genetic variants on clinical outcomes was tested: a lowering of bone turnover markers, an increase of bone quality (QUI measurements) and an increase in bone mineral density. The CTX was shown to be influenced by a number of *1b haplotype copies, which was shown also to increase the raloxifene species plasma concentration, that most probably caused a higher decrease in CTX during treatment. A statistical significance was observed also in change of BMD-FN regarding *SLCO1B1 c.*388A > G polymorphism and *1a haplotype but due to the small sample size, only modest changes in BMD-FN in one year and the inherent measurement error by DXA [51] the direction could not be determined. Additionally, no influence of *SLCO1B1* and *SLCO1B3* genetic variants on HOL, HDL, LDL and TG was observed.

Beside the influence of *SLCO1B1* and *SLCO1B3* genetic variants on raloxifene pharmacokinetics and pharmacodynamics the influence of studied polymorphisms on the probability of raloxifene adverse effects was also evaluated. The statistical analysis did not establish any correlation of studied polymorphisms with the probability of raloxifene adverse effects because the study was not designed for this purpose and number of patients was too low. Further studies with higher number of patients are needed to discover the possible associations of genetic polymorphisms with raloxifene adverse effects.

Our results indicate that a significant part (up to 20%) of the observed high inter-individual variability in pharmacokinetics and pharmacodynamics can be explained by a genetic influence of the *SLCO1B1 c.*388A > G polymorphism. Therefore, pharmacogenetics of *SLCO1B1* could be taken into account to individualize raloxifene therapy.

The main limitation of the current *in vivo* study is a rather small number of the enrolled participants. Additionally, the duration of our study should be prolonged due to very minimal changes in BMD observed in one year. Other pharmacodynamic parameters and concentrations of raloxifene species should also be monitored in such prolonged study. Moreover, multiple comparisons may be the cause for the reported positive associations. As such, our results are exploratory in nature and should be interpreted as hypothesis generating and confirmed by the accomplishment of a lager study. However, it is our opinion that the present *in vivo* study in connection with the *in vitro* data allows to make stated interpretations.

## Conclusions

In conclusion, this is the first study where *in vitro* research of uptake transporters for raloxifene and its metabolites was performed and also the first study to assess the influence of *SLCO1B1* and *SLCO1B3* polymorphisms on pharmacokinetics and pharmacodynamics of raloxifene. It was shown that raloxifene interacts with OATP1B1 and M2 and M3 interact with both tested uptake transporters. Furthermore, despite the small number of study participants, the *in vivo* evidence showed the influence of *SLCO1B1* 388A > G polymorphism and *1b haplotype on pharmacokinetics and pharmacodynamics of raloxifene.

## Abbreviations

BALP: Serum bone-specific alkaline phosphatase; BMD: Bone mineral density; BUA: Broadband ultrasound attenuation; CHO: Chinese Hamster Ovary; CTX: Serum C-terminal telopeptide fragments of type I collagen; E-3-S: Estrone-3-sulfate; ICG: Indocyanine green; M1: Raloxifene-6-β-glucuronide; M2: Raloxifene-4'-β-glucuronide; M3: Raloxifene-6,4'-diglucuronide; OATP: Organic anion-transporting polypeptide; OC: Serum osteocalcin; PD: Pharmacodynamic parameter; QUI: Quantitative ultrasound index; QUS: Quantitative ultrasound parameter; SLCO: Solute carrier organic anion transporter gene; SOS: Heel speed of sound.

## Competing interests

The authors declare that they have no competing interests.

## Authors’ contributions

TTL drafted the manuscript, performed the experiments on transfected cell lines, performed the statistical analysis. BS performed the experiments on transfected cell lines, reviewed the manuscript. JM participated in the design of the study, performed clinical study, reviewed the manuscript. AM participated in the design of the study, performed clinical study, reviewed the manuscript. JT measured plasma concentrations of raloxifene species, reviewed the manuscript. AZ performed clinical study, reviewed the manuscript. BO participated in the design of the study, performed clinical study, performed genotyping, reviewed the manuscript. All authors read and approved the final manuscript.
